# MDTips: a multimodal-data-based drug–target interaction prediction system fusing knowledge, gene expression profile, and structural data

**DOI:** 10.1093/bioinformatics/btad411

**Published:** 2023-06-28

**Authors:** Xiaoqiong Xia, Chaoyu Zhu, Fan Zhong, Lei Liu

**Affiliations:** Institutes of Biomedical Sciences, Fudan University, No. 131, Dong An Road, Shanghai, Shanghai 200032, China; Intelligent Medicine Institute, Shanghai Medical College, Fudan University, 131 Dongan Road, Shanghai, Shanghai 200032, China; Intelligent Medicine Institute, Shanghai Medical College, Fudan University, 131 Dongan Road, Shanghai, Shanghai 200032, China; Institutes of Biomedical Sciences, Fudan University, No. 131, Dong An Road, Shanghai, Shanghai 200032, China; Intelligent Medicine Institute, Shanghai Medical College, Fudan University, 131 Dongan Road, Shanghai, Shanghai 200032, China; Shanghai Institute of Stem Cell Research and Clinical Translation, Shanghai, Shanghai 200120, China

## Abstract

**Motivation:**

Screening new drug–target interactions (DTIs) by traditional experimental methods is costly and time-consuming. Recent advances in knowledge graphs, chemical linear notations, and genomic data enable researchers to develop computational-based-DTI models, which play a pivotal role in drug repurposing and discovery. However, there still needs to develop a multimodal fusion DTI model that integrates available heterogeneous data into a unified framework.

**Results:**

We developed MDTips, a multimodal-data-based DTI prediction system, by fusing the knowledge graphs, gene expression profiles, and structural information of drugs/targets. MDTips yielded accurate and robust performance on DTI predictions. We found that multimodal fusion learning can fully consider the importance of each modality and incorporate information from multiple aspects, thus improving model performance. Extensive experimental results demonstrate that deep learning-based encoders (i.e. Attentive FP and Transformer) outperform traditional chemical descriptors/fingerprints, and MDTips outperforms other state-of-the-art prediction models. MDTips is designed to predict the input drugs’ candidate targets, side effects, and indications with all available modalities. Via MDTips, we reverse-screened candidate targets of 6766 drugs, which can be used for drug repurposing and discovery.

**Availability and implementation:**

https://github.com/XiaoqiongXia/MDTips and https://doi.org/10.5281/zenodo.7560544.

## 1 Introduction

Developing a new drug always takes 10–15 years and costs about 0.8–1.5 billion dollars, but it faces a considerable risk of failure (Paul *et al.* 2010). To alleviate this dilemma, a novel drug development strategy, drug repurposing (Pushpakom *et al.* 2019), was proposed to discover new indications for existing drugs. Large-scale and reliable prediction of drug–target interactions (DTIs) will substantially facilitate drug repurposing and development.

With the development of AI techniques and the accumulation of large-scale biomedical data, deep learning (DL)-based DTI prediction models exhibit a great advantage over traditional computational methods such as molecular docking (Forli *et al.* 2016) and machine learning (Bagherian *et al.* 2021), thus, attracting increasing research attention to tackle the DTI prediction problem (Du *et al.* 2022). Generally, the DL-based models for DTI prediction can be classified into two types: single and multimodality models.

The single models for DTI focused on learning representations of drugs and targets from the specific modality. Then the concatenate representations are fed to a fully connected network to predict the DTI probability. Advanced DL techniques, such as convolutional neural networks (CNN) (Öztürk *et al.* 2018), Transformer (Huang *et al.* 2022), and graph neural networks (GNN) (Zhao *et al.* 2021) have motivated their application in DTI prediction models. They take the 1D sequential strings, 2D graphs or image grids (de Souza *et al.* 2022), or 3D structures (Yazdani-Jahromi *et al.* 2022) of drugs and targets as input and automatically learn representation vectors from the complex data types. In addition, several models are constructed from a genomics perspective, representing the features of drugs and targets with gene expression profiles (Xie *et al.* 2018, Shao *et al.* 2020, Zhong *et al.* 2022). Despite these promising developments, they have several limitations. First, large amounts of known drug–target pairs (DTPs) must be used to train DL-based DTI models, while the labeled data volume is always limited. Second, they face cold-start problems, where the model accuracy decreases when predicting the interaction of a novel drug without knowing any target information (Nguyen *et al.* 2022).

More recently, multimodality models have rapidly progressed for DTI prediction based on single-modality-based models by integrating heterogeneous data into a unified framework. The heterogeneous data, including drug–target, drug–disease, and drug–drug (Zhu *et al.* 2019) interactions, formed a complicated biological network where nodes are drugs and targets, while edges are interactions, and node attributes are structural information or/and gene expression signatures of drugs and targets. Multimodal fusion, which has boosted the performance of many classical problems (e.g. visual question-answering) (Xue and Marculescu 2022), is employed to integrate heterogeneous information from networks and automatically extract features of drugs and targets to facilitate further DTI prediction (Nguyen *et al.* 2022). Many models integrate diverse entities (e.g. drug, target, and disease) and edge types in the heterogeneous network or knowledge graph by GNN (Li *et al.* 2023), network-based methods (Tian *et al.* 2022), and knowledge graph embedding (KGE) methods (Li *et al.* 2022). Furthermore, to incorporate drugs and targets’ structural information and networks, these works design the protein sequence encoder and drug structure encoder to extract the initial features for targets and drugs and then fuse structural information and networks by GCN-based interaction (Wang *et al.* 2022), joint representation framework based on heterogeneous networks (Zhou *et al.* 2021), neural factorization machine (Ye *et al.* 2021), and shared unit (Ma *et al.* 2023). Such models can automatically learn representations of drugs and targets from structural information and heterogeneous network in an end-to-end manner, thus outperforming single-modality models.

Despite these promising developments in multimodality models, there still exist two shortcomings: (i) these models focus on fusing features from at most two modalities of structural information and heterogeneous network, ignoring gene expression profiles, which represent biological responses and changes in cellular processes to diverse perturbations (e.g. compound and gene knockout) (Tanoli *et al.* 2021). In addition, they lack a comprehensive evaluation of each modality’s contribution; (ii) these models are tailored for predicting DTIs, ignoring other related drug information such as side effects and indications, which is essential for drug repurposing.

To address the above issues, we developed MDTips by integrating the knowledge graphs, gene expression profiles, and structural information of drugs/targets. Specifically, for structural information, we use Attentive FP and Transformer to learn representation vectors from 2D drug molecular graphs and 1D target amino acid sequences (AAS), respectively. We apply fully connected feed-forward networks (FC) for gene expression signatures to extract high-dimensional features of drugs and targets, respectively. For knowledge graphs, we employ ConvE (Dettmers *et al.* 2018) to learn the embeddings of entities and relations. This way, we can fuse all data modalities by concatenating feature vectors and feed the fused representation to an FC module to calculate the interaction probability. Further, the framework enables a comprehensive evaluation of each modality. From the application’s perspective, it can efficiently utilize KG and available modalities to predict the input drug/compound’s potential targets, indications, and side effects.

To summarize, MDTips differs from previous multimodality models by (i) fusing three data modalities of drugs and targets, (ii) enabling comparison of modality’s contribution, (iii) providing a pretrained model for predicting multiple drug information.

## 2 Materials and methods

### 2.1 Datasets

This study uses a comprehensive biological KG: Drug Repurposing Knowledge Graph (DRKG) (Ioannidis 2020), which includes 97 238 entities belonging to 13 entity types and 5 874 261 triplets belonging to 107 edge types. We extract 88 439 DTPs from DRKG as DTI task-related dataset DTP_KS_. The remaining triplets with multiple relations (e.g. drug–drug, drug–disease, and gene–disease) are supporting knowledge $\;{\mathrm{KG}}_{\mathrm{support}}$. Drugs’ SMILES strings are collected from DrugBank (Wishart *et al.* 2018) and then converted into canonical SMILES using the RDKit package. Targets’ AAS are collected from UniProt. We extract 24 418 DTPs with all three modalities (i.e. KG, graph/sequence, and gene expression signature) from ${\mathrm{DTP}}_{\mathrm{KS}}$ to get the subset ${\mathrm{DTP}}_{\mathrm{KSE}}$ (Table 1). The gene expression signatures used to measure consensus transcriptional response to perturbations of 978 genes are downloaded from consensus signatures for LINCS L1000 perturbations (Daniel Himmelstein and Baranzini 2016). All the DTPs in ${\mathrm{DTP}}_{\mathrm{KS}}$ or ${\mathrm{DTP}}_{\mathrm{KSE}}$ are positive samples. Negative samples are generated by combining *n* drugs and *m* targets into n×m pairs and filtering all positive samples. We under-sampled negative samples with a positive-to-negative sampling ratio of 1:2. The positive and negative samples generated from ${\mathrm{DTP}}_{\mathrm{KS}}$/${\mathrm{DTP}}_{\mathrm{KSE}}$ are mixed to form the experiment dataset ${\mathrm{Dataset}}_{\mathrm{KS}}$/${\mathrm{Dataset}}_{\mathrm{KSE}}$.

**Table 1.** Brief statistics of ${\mathrm{DTP}}_{\mathrm{KS}}$ and ${\mathrm{DTP}}_{\mathrm{KSE}}$.

Dataset	Interaction type	DTP	Drug	Target
${\mathrm{DTP}}_{\mathrm{KS}}$	–	88 439	6766	8089
${\mathrm{DTP}}_{\mathrm{KSE}}$	–	24 418	857	2108
	Inhibit/downregulate	16 810	816	1721
	Activate/upregulate	7608	602	917

### 2.2 The workflow of MDTips

**Figure 1.**
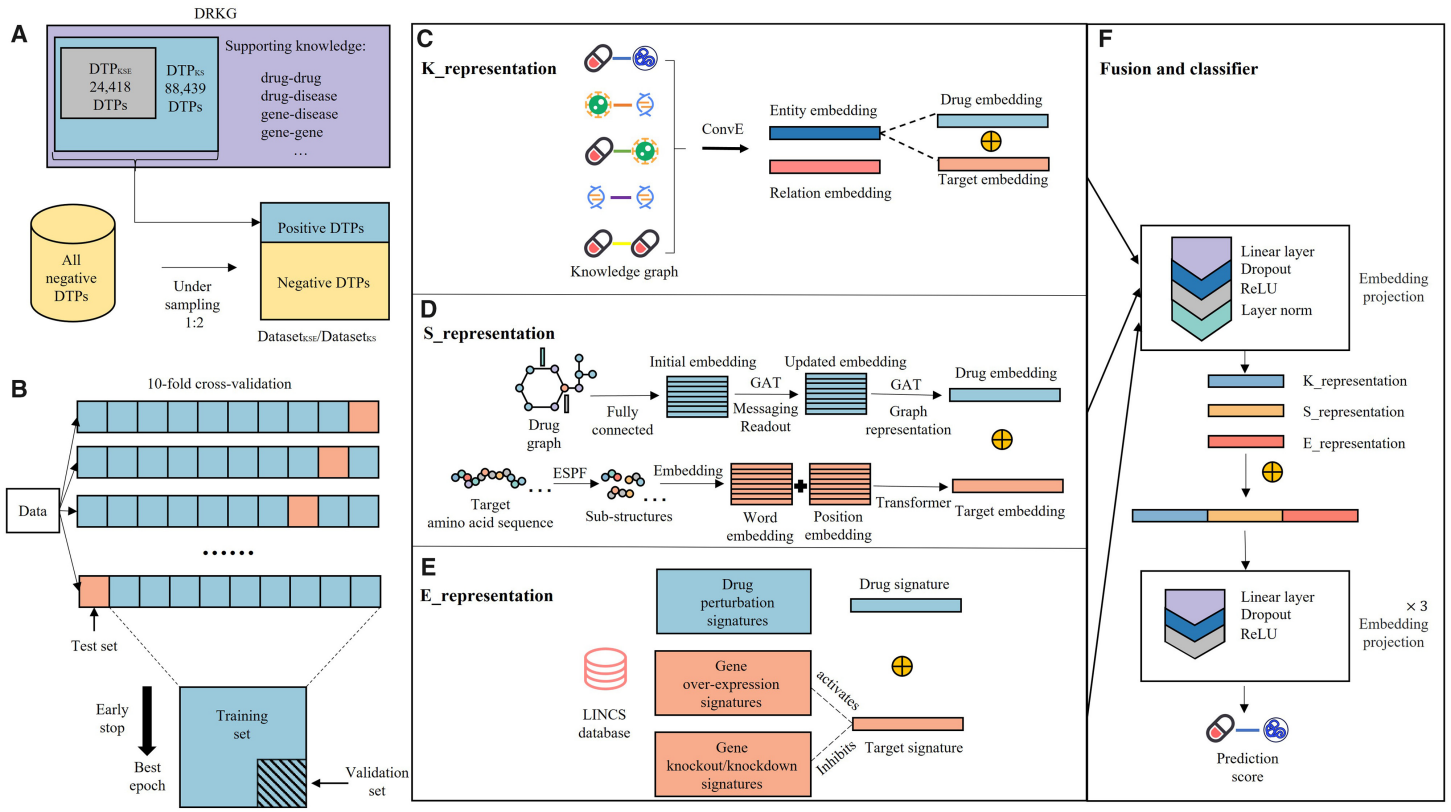
The pipeline of building MDTips. (A) The data space of ${\mathrm{DTP}}_{\mathrm{KS}}$ and ${\mathrm{DTP}}_{\mathrm{KSE}}$, negative samples are generated by the under-sampling strategy. Positive DTPs and negative samples are mixed to form dataset_KS and dataset_KSE. (B) The 10-fold cross-validation strategy used in this study. The data were randomly divided into a training set, a validation set, and a test set with a ratio of 8:1:1. The schematic workflow of (C) K_representation, (D) S_representaion, (E) E_representation, and (F) multimodal fusion and classifier.

MDTips is a binary classification model. The input is the DTP (d, t), and the output is *y*∈{0, 1}, when *y *=* *1, indicates an interaction between the input drug d and the target t. MDTips consists of two main components: (i) representation modules that learn the representations of d and t; (ii) a fusion and decoder module that integrates representations generated by each representation module and predicts the label of (d, t) (Fig. 1). MDTips considers all the modalities of drugs and targets in the existing datasets. ${\mathrm{Dataset}}_{\mathrm{KS}}$ contains two modalities (i.e. KG, graph/sequence) and ${\mathrm{Dataset}}_{\mathrm{KSE}}$ contains all the three modalities (i.e. KG, graph/sequence, and expression signature). For DTPs in ${\mathrm{Dataset}}_{\mathrm{KSE}}$, we design three representation modules. Those are K_representation (K), S_representation (S), and E_representation (E), which learn drug/target representations based on KGs, graphs/sequences, and gene expression signatures, respectively. We then fuse the learned drug/target representations and feed the fused embedding into a four-layer perceptron neural network. Finally, the prediction score of DTI can be evaluated. The evaluation metrics and experiment details are presented in Supplementary data.

#### 2.2.1 S_representation

##### 2.2.1.1 Attentive FP for drug representation

Drugs are converted to molecular graphs, where nodes represent atoms, and edges represent bonds. We construct vectors to represent the chemical features of nodes (e.g. atom type) and edges (e.g. conjugated bond) using RDKit and DGL-LifeSci packages. Concretely, formal charge and radical electron number are encoded as integers, and other features are encoded as one-hot vectors. This approach generates vectors with a length of 39 for nodes and 11 for edges (Supplementary Tables S1 and S2). The molecular graphs with node and edge features are fed into Attentive FP to learn drug representations.

Attentive FP introduces an attention mechanism to GNNs, allowing a target atom to focus on its neighborhood’s most relevant “messages” (Xiong *et al.* 2020). The atom representation is updated in the messaging and readout phases, which are formulated as follows: where v is the target atom, and u is the neighbor atom of v. At the k-1 layer, $h_{u}^{k-1}$ and $h_{v}^{k-1}$ are the representation vectors of u and v. $M^{k-1}$ is the message function, which uses the graph attention network (GAT) (Supplement data) (Veličković *et al.* 2017). In the readout phase, the current representation $h_{v}^{k}$ is calculated by applying the update function: gated recurrent unit ${\mathrm{GRU}}^{k-1}$ (Supplement data) on the previous representation: $h_{v}^{k-1}$ and the attention context $C_{v}^{k-1}$. After obtaining updated node representations, we then compute graph-level representations out of node features using the method introduced by Xiong *et al.* (2020).


(1) $$\mathrm{Messaging}:\;C_{v}^{k-1}=\sum_{u\in N(v)}M^{k-1}\left(h_{u}^{k-1},h_{v}^{k-1}\right)$$

(2) $$\mathrm{Readout}:\;h_{v}^{k}={\mathrm{GRU}}^{k-1}\left(C_{v}^{k-1},\;h_{v}^{k-1}\right)$$

##### 2.2.1.2 Transformer for target representation

Explainable substructure partition fingerprint (Huang *et al.* 2019) is used to decompose targets’ AAS into a discrete set of moderate-sized substructures, and the predefined subsequences vocabular set is $S=\{s_{1},s_{2},\ldots {,s}_{4114}\}$. To get inspiration from natural language processing, we use transformer model (Vaswani *et al.* 2017) for target representation learning.

For an input target T, we decompose the AAS to a sequence of substructures $T=\{t_{1},t_{2},\ldots {,t}_{l}\}$, where $t_{i}\in S$. We then map T to a representations vector $Z=(z_{1},z_{2},\ldots {,z}_{l})$, where $z_{i}$ is the substructure index in S for $t_{i}$. The padding operation is used to unify the length of substructure sequences, and the maximum length m=545. To capture the positional information of sequences, we define a positional vector $P=(p_{1},p_{2},\ldots ,p_{l})$, where $p_{1}=i$. We then generate the target substructure embedding $E_{\mathrm{seq}}{\in R}^{m*d}$ and positional embedding $E_{\mathrm{pos}}{\in R}^{m*d}$ using the word embedding method, where d=64. The target representation $E\in R^{m*d}$ is formulated as:


(3) $$E=E_{\mathrm{seq}}+E_{\mathrm{pos}}$$

The embedding E is fed to a transformer encoder including two sublayers, i.e. a multihead self-attention layer (m_Atten) (Supplementary data) and a fully connected feed-forward network (FC). Firstly, m_Atten is used to compute a new target representation by considering contextual information of sequences. Then, the updated representation is fed into FC, followed by dropout, layer normalization, and residual connection modules. The process is formulated as:


(4) $$E_{\mathrm{update}}=m\_\mathrm{Atten}(E)$$

(5) $$\mathrm{FC}:E_{\mathrm{update}}=\mathrm{LayerNorm}\left(E+\left(E_{\mathrm{update}}W+b\right)\right).$$

#### 2.2.2 K_representation

The knowledge graph K is a multirelational graph that comprises a set of triples (h, r, t), where h, r, and t represent the head entity, relation, and tail entity, respectively. We use a KGE model ConvE (Dettmers *et al.* 2018) to learn representations for all entities and relations in K. The embedding vectors of h, r, and t are randomly initialized as $e_{h}\in R^{k}$, $r_{r}\in R^{k}$, and $e_{t}\in R^{k}$, where k is the embedding dimension of entity and relation. Then the interaction score of (h, r, t) is calculated with scoring function $\varphi_{r}(e_{h},e_{t})$, which is defined as: where ReLU is the rectified linear units, $\bar{e_{h}}\in R^{k_{w}*k_{h}}$ and $\bar{r_{r}}\in R^{k_{w}*k_{h}}$ are 2D reshaping of $e_{h}$ and $r_{r}$, where $k=k_{w}{*k}_{h}$. The 2D convolutional layer with filters ω is applied to the concatenated matrix $\left[\bar{e_{h}},\bar{r_{r}}\right]$. Binary cross-entropy loss (BCELoss) is applied to update the initial embedding and model parameters.


(6) $$\varphi_{r}\left(e_{h},e_{t}\right)=\mathrm{ReLU}\left(\mathrm{vec}\left(f\left(\left[\bar{e_{h}},\bar{r_{r}}\right]*\omega \right)\right)W\right)e_{t}$$

#### 2.2.3 E_representation

For each DTP in ${\mathrm{DTP}}_{\mathrm{KSE}}$, the drug d is represented as the molecule perturbated signature, and the target t is represented as the gene knockdown/overexpression perturbated signature if the drug d inhibits/activates the target t. Specifically, the representation of drug d and target t is defined as: where $M_{D}\in R^{n_{D}*978}$, $M_{\mathrm{OE}}\in R^{n_{\mathrm{OE}}*978}$, $M_{\mathrm{XPR}}\in R^{n_{\mathrm{XPR}}*978}$ are consensus signature datasets, and D, OE, XPR are perturbation sets of drugs, gene overexpression, and gene knockdown, respectively.


(7) $$E_{d}=M_{D}\left[d\right],\;d\in D$$

(8) $$E_{t}=M_{\mathrm{OE}}\left[t\right],\;t\in \mathrm{OE},\;\mathrm{if}\;\mathrm{interaction}=\mathrm{activate}$$

(9) $$E_{t}=M_{\mathrm{XPR}}\left[t\right],\;t\in \mathrm{XPR},\;\mathrm{if}\;\mathrm{interaction}=\mathrm{inhibit}$$

#### 2.2.4 Fusion and decoder module

The representations of drugs and targets are learned by K, S, and E modules. We define these learned representations of drug $d_{i}$ as $d_{ik}$, $d_{is}$, $d_{ie}$, and target $t_{j}$ as $t_{jk}$, $t_{js}$, $t_{je}$. ${\mathrm{score}}_{ij}$ is the interaction score of the ${\mathrm{DTP}}_{ij}$. We first map each modality representation of ${\mathrm{DTP}}_{ij}$ into a latent feature space through FC with a Dropout and an ReLU activation, followed by layer normalization (Lei Ba *et al.* 2016). For example, the S representation of ${\mathrm{DTP}}_{ij}$ is formulated as: where $h_{s}$ is ${\mathrm{DTP}}_{ij}$’s hidden state of the S representation. W and b are learnable parameters in the training process. The dropout rate is set at 0.1. The same operations are applied to K and E representations. We then concatenate $h_{k}$, $h_{s}$, and $h_{e}$ and feed into a four-layer perceptron neural network, of which three hidden layers include 1024, 1024, and 512 neural units, separately. We use the Sigmoid function in the final layer to map the output value to [0,1]. The BCELoss function is used to train the model by back-propagating the error and updating all parameters of the model in an end-to-end manner.


(10) $$h_{s}=\mathrm{ReLU}\left(\mathrm{Dropout}\left(\mathrm{Concat}\left(d_{is},t_{js}\right)W_{s}+b_{s}\right)\right)$$

(11) $$h_{s}=\mathrm{LayerNorm}(h_{s})$$

## 3 Results

### 3.1 Comparison with other S_representation modules

**Figure 2.**
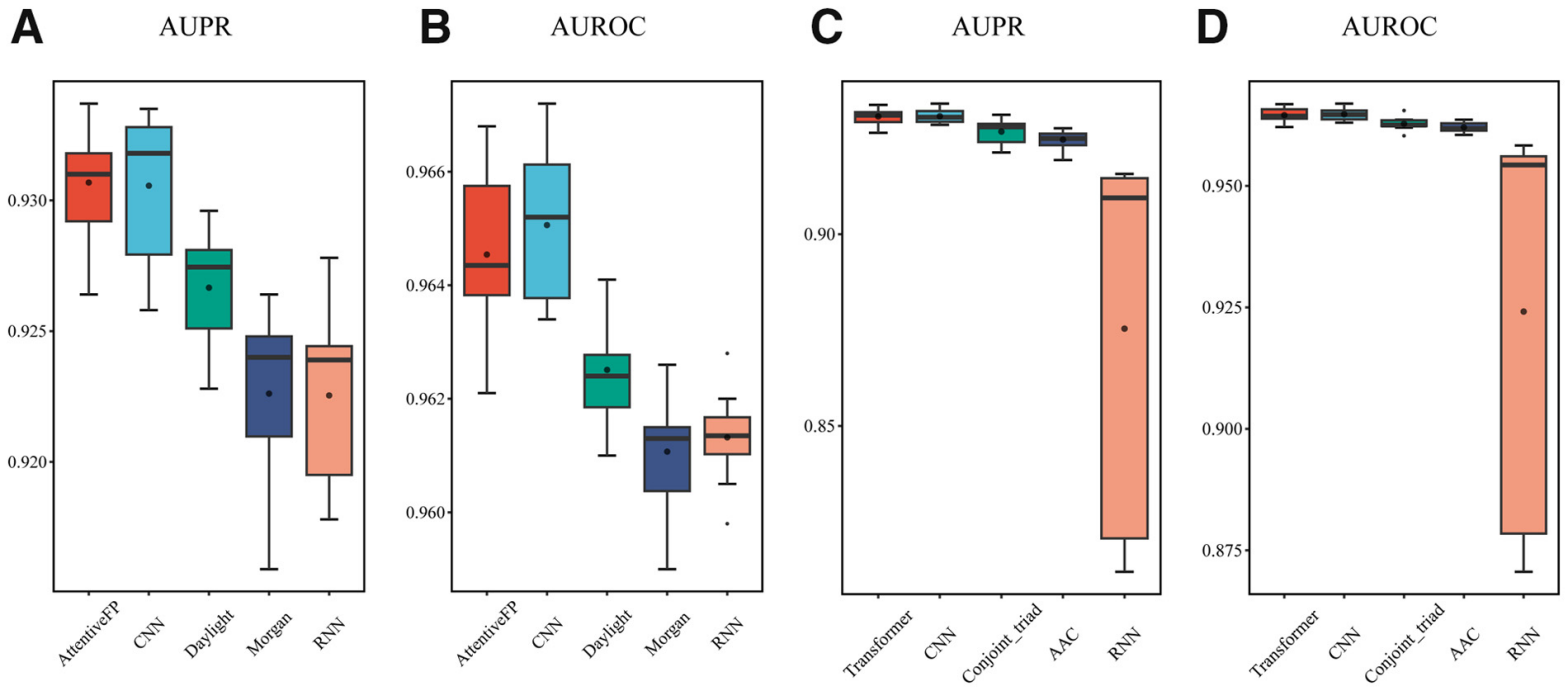
Evaluation performance of S_representations on ${\mathrm{Dataset}}_{\mathrm{KS}}$. (A, B) compares Attention FP with other drug encoders. (C, D) Compares Transformer with other target encoders. The black dot in the boxplot shows the average value, and the horizontal line shows the median.

Our S_representation module includes Attentive FP for drug representation and Transformer for target representation. To explore the impacts of different drug/target encoders on MDTips, we first compared drug encoders: Attentive FP with two molecular fingerprint-based methods: Morgan and Daylight, and two sequence-based methods: CNN and RNN. We then compared the target encoders: Transformer with two target descriptors: amino acid composition (AAC) up to 3-mers (Du *et al.* 2014) and conjoint triad features (Shen *et al.* 2007), and two sequence-based methods: CNN and RNN. We conducted all the experiments on ${\mathrm{Dataset}}_{\mathrm{KS}}$. Attentive FP is a superior drug encoder compared to Daylight, Morgan, and RNN and comparable to CNN (Fig. 2A). Its higher average AUPR value (primary metric) and interpretability (Xiong *et al.* 2020) make it useful for guiding chemists in structural optimization for target interaction. Transformer performs better than other target encoders (Fig. 2C and D). For example, the AUPR and area under the receiver operating characteristics (AUROC) of Attentive FP are significantly higher than that of Morgan, with an increase of 0.8% (*P *=* *.002) and 0.3% (*P *=* *.003), respectively. The AUPR and AUROC of the Transformer are significantly higher than AAC, with an increase of 0.6% (*P *=* *.002) and 0.25% (*P *=* *.009), respectively.

In conclusion, Attentive FP and Transformer outperform traditional chemical descriptors/fingerprints and biological features obtained from databases.

### 3.2 Performance on Dataset_KSE_

**Figure 3.**
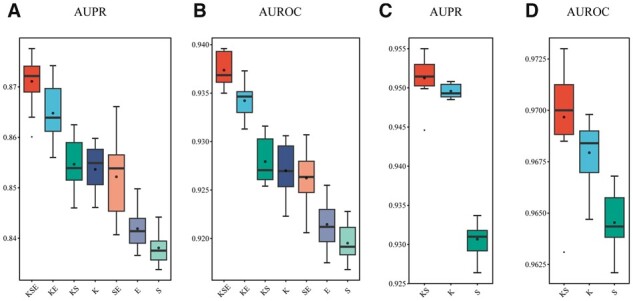
Evaluation performance of multimodal fusion. (A, B) Compares three single-modality models: K, S, E, and the fused ones: KS, KE, SE, and KSE on ${\mathrm{Dataset}}_{\mathrm{KSE}}$. (C, D) Compares two single-modality models: K and S, and the fused KS on ${\mathrm{Dataset}}_{\mathrm{KS}}$.

We verified the effectiveness of three single modalities: K, S, E, and the fused ones: KS, KE, SE, and KSE on ${\mathrm{Dataset}}_{\mathrm{KSE}}$. KSE performs best among all seven models (Fig. 3A and B). The AUPR and AUROC values of KSE are significantly higher than that of other models (*P *<* *.05). The KSE, KE, KS, and K models that utilize KG perform better than those without KG (SE, E, and S). KG-driven DTI models not only utilize the interaction between drugs and targets but also incorporate other information from the large-scale triples in KG (e.g. drug–drug, drug–disease, and gene–disease), thus significantly improving DTI models’ performance. Additionally, we observed that fused models significantly outperform single-modality models. For example, SE is better than S and E; KE is better than K and E. The result is consistent with an intuition that different modalities provide information from different aspects and improve performance. Multimodal fusion learning can fully consider the importance of each modality. Additionally, we found that each model has a low standard deviation of AUPR and AUROC, demonstrating each model’s robustness.

### 3.3 Performance on Dataset_KS_

We verified the effectiveness of two single modalities K and S, and the fused KS on ${\mathrm{Dataset}}_{\mathrm{KS}}$. The KS model achieves the best performance (AUPR = 0.951 ± 0.003 and AUROC = 0.970 ± 0.003). The AUPR and AUROC of KS are significantly higher than that of S (AUPR: *P *=* *.002 AUROC: *P *=* *.006), but they do not have a significant difference between KS and K (AUPR: *P *=* *.064, AUROC: *P *=* *.064) (Fig. 3C and D). These results suggest that the KG plays a pivotal role in the DTI model, and structural information of drugs/targets is only subordinate when KG is provided.

### 3.4 The framework of MDTips

**Figure 4.**
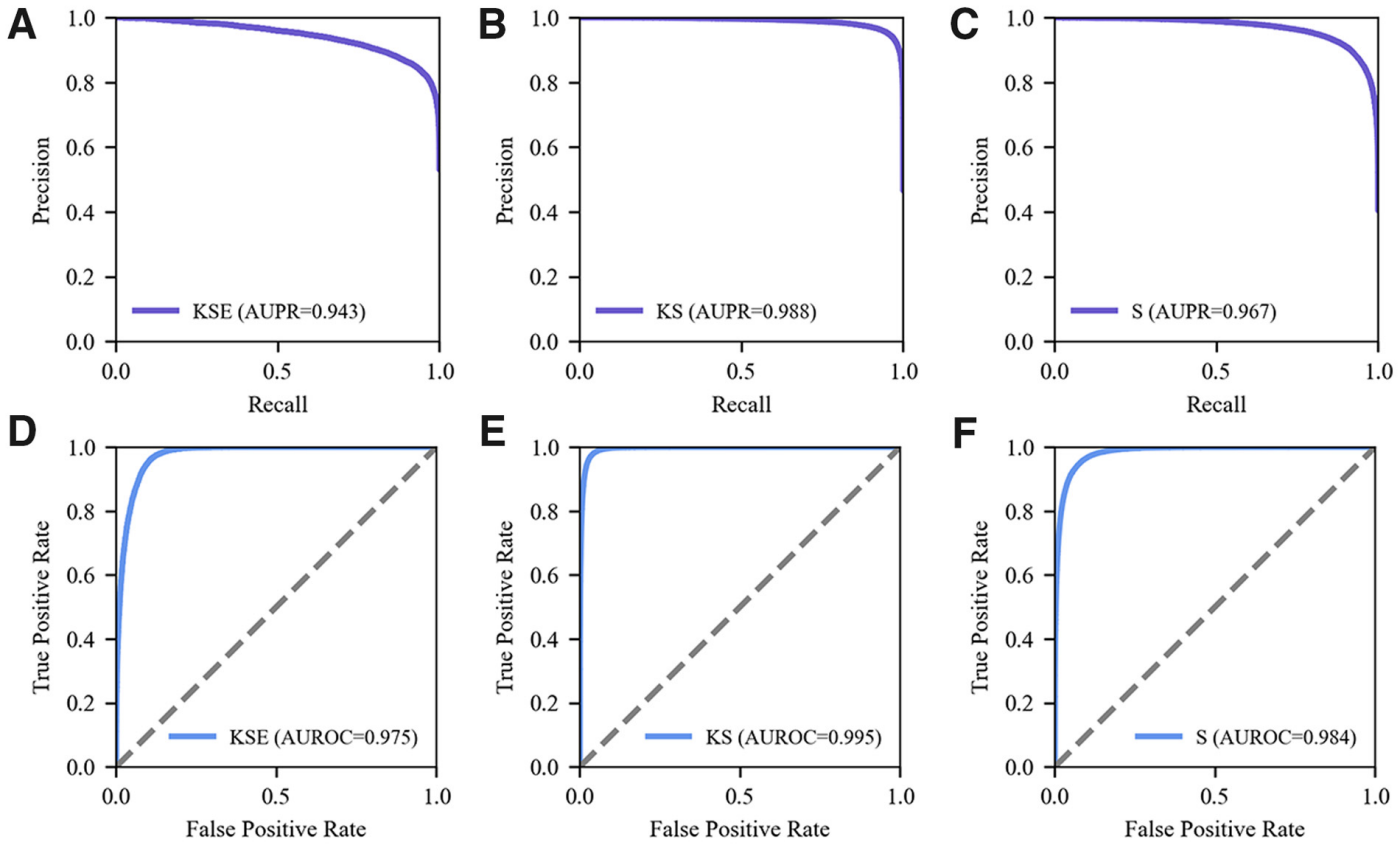
Predictive performance of MDTips framework on ${\mathrm{Dataset}}_{\mathrm{KSE}}$ and ${\mathrm{Dataset}}_{\mathrm{KS}}$. (A, D) PR and ROC curves of KSE on ${\mathrm{Dataset}}_{\mathrm{KSE}}$, respectively. (B, E) PR and ROC curves of KS on ${\mathrm{Dataset}}_{\mathrm{KS}}$, respectively. (C, F) PR and ROC curves of S on ${\mathrm{Dataset}}_{\mathrm{KS}}$, respectively.

The results indicate that KS and KSE perform best on ${\mathrm{Dataset}}_{\mathrm{KS}}$ and ${\mathrm{Dataset}}_{\mathrm{KSE}}$, respectively. Since multimodal fusion efficiently integrates the information from KGs, graphs/sequences of drugs/targets, and gene expression signatures for DTI prediction, we trained two fusion prediction models: KS and KSE, on ${\mathrm{Dataset}}_{\mathrm{KS}}$ and ${\mathrm{Dataset}}_{\mathrm{KSE}}$ by using all available modalities. We also trained the S model for drugs with only structural information on ${\mathrm{Dataset}}_{\mathrm{KS}}$. The high AUPR and AUROC values demonstrate that these prediction models are powerful enough to detect the true DTIs labeled in the datasets (Fig. 4). The DTP scores predicted by KSE, KS, and S models strongly correlate (Spearman *r *>* *0.8 and *P *<* *.001), indicating relatively high consistency.

Based on the above results, we constructed a comprehensive drug-centric prediction framework named MDTips, containing the KSE, KS, S, and K models. MDTips is an open system that utilizes all available modalities to predict candidate targets of compounds, and it employs the input SMILES to conduct a comprehensive database search for matching models. In cases where three available modalities are associated with the input SMILES, MDTips deploys the KSE model. For novel compounds that lack any prior knowledge, the S_model is utilized for predicting their candidate targets (Supplementary Fig. S1). Additionally, drug-related information (e.g. side effects and indications) can be predicted by loading the pretrained K-model or re-training the K model on the combination of the predicted DTIs and the previous KG. We implemented all prediction processes with a few code lines, facilitating parallel prediction for massive drugs/compounds.

### 3.5 Comparison with other methods on Dataset_KS_

We compared MDTips with six DTI prediction methods. There are two knowledge-based: KGE_NFM (Ye *et al.* 2021) and DRKG (Ioannidis 2020); a sequence-based: DeepDTA (Öztürk *et al.* 2018); two graph-based: CPI-GNN (Tsubaki *et al.* 2019) and GraphDTA (Nguyen *et al.* 2021); a 3D structure-based: AttentionSiteDTI (Yazdani-Jahromi *et al.* 2022). We first compared ${\mathrm{Dataset}}_{\mathrm{KS}}$ with the custom-built datasets of the six models, and found ${\mathrm{Dataset}}_{\mathrm{KS}}$ covers the most extensive positive DTIs (Table 2). So, we compared MDTips with the other six models (Supplement data) using ${\mathrm{Dataset}}_{\mathrm{KS}}$.

**Table 2.** Comparisons of feature, scale, and functionality of MDTips with other methods.

Model	Modalities			Statistics			Featurization		Functionality of predicting
	K	E	S	D/C	Targets	DTPs	Drug	Target	
MDTips	√	√	√	6766 + unlimited^a^	8089	88 439	Attentive FP	Transformer	DTIs, related drugs, indications, side effects, pharmacologic classes
KGE_NFM (Ye *et al.* 2021)	√		√	6214	3442	26 051	Morgan fingerprints	CTD descriptors	DTIs
DRKG (Ioannidis 2020)	√	√		6766	8089	88 439	KGE	KGE	Entities
DeepDTA (Öztürk *et al.* 2018)			√	2111	229	–	CNN	CNN	Drug–target binding affinity
CPI-GNN (Tsubaki *et al.* 2019)			√	1434	2504	4000	GNN	CNN	Drug–target binding affinity
GraphDTA (Nguyen *et al.* 2021)			√	2116	229	–	GNN	CNN	Drug–target binding affinity
AttentionSiteDTI (Yazdani-Jahromi *et al.* 2022)			√	989 383	8536	39 747	TAGCN	TAGCN	DTIs

^a^MDTips can predict drug-related information open to unlimited compounds with all available modalities. DeepDTA and GraphDTA are regression models, and the label of DTP is a constant value: a KIBA score that cannot be discretized with a threshold (positive or negative). DeepDTA, GraphDTA, and CPI-GNN were originally designed for predicting drug–target binding affinity. Here, we used the models for predicting drug–target interactions.

K, KGs; E, expression profiles; S, graphs/sequences; D/C, drugs/compounds.

MDTips and all six models achieve relatively high predictive performance (Table 3). Among these, MDTips achieves the best performance (AUPR = 0.951 ± 0.003), significantly higher (*P *=* *.002) than that of the second-best model KGE_NFM (AUPR = 0.947 ± 0.002). The KG-based models MDTips, KGE_NFM, and DRKG performed better than the structure/graph/sequence-based models DeepDTA, CPI-GNN, GraphDTA, and AttentionSiteDTI. This result is consistent with the previous results that KG can significantly improve the performance of DTI models by integrating other data information. Possible causes of AttentionSiteDTI does not perform well on the ${\mathrm{Dataset}}_{\mathrm{KS}}$ are: (i) multiple different-effect DTIs exist, such as binding, upregulated/downregulated, and inhibit/activate. (ii) We use the AlphaFold2 predicted 3D structures of targets if they do not have experimentally determined structures in the Protein Data Bank (PDB), which may introduce errors in the model.

**Table 3.** The performance of MDTips and other methods on ${\mathrm{Dataset}}_{\mathrm{KS}}$.

Model	Data type	AUPR	AUROC
MDTips	KG + sequence	**0.951** ± **0.003**	0.970 ± 0.003
KGE_NFM	KG + sequence	0.947 ± 0.002	**0.973** ± **0.001**
DRKG	KG	0.949 ± 0.002	0.967 ± 0.002
DeepDTA	Sequence	0.930 ± 0.003	0.964 ± 0.002
CPI-GNN	Sequence	0.929 ± 0.003	0.963 ± 0.001
GraphDTA	Sequence	0.928 ± 0.002	0.962 ± 0.001
AttentionSiteDTI	3D structure	0.864 ± 0.027	0.923 ± 0.026

The best performer and the second-best performer are highlighted in bold and underlined, respectively.

### 3.6 Performance of MDTips with different under-sampling ratios

**Figure 5.**
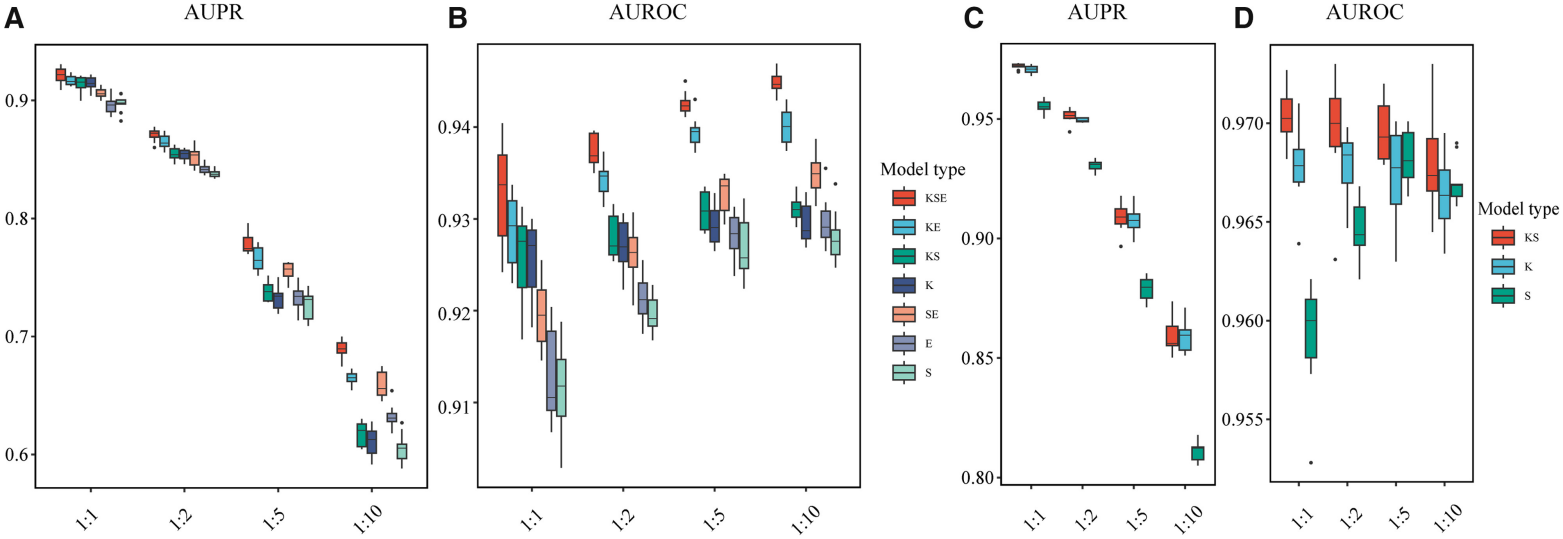
(A, B) Evaluation performance of multimodal fusion on Dataset_KSE with four different positive-to-negative sampling ratios of 1:1, 1:2, 1:5, and 1:10. (C, D) Evaluation performance of multimodal fusion on Dataset_KS with four different positive-to-negative ratios of 1:1, 1:2, 1:5 and 1:10.

The sampling ratio of ${\mathrm{Dataset}}_{\mathrm{KS}}$ and ${\mathrm{Dataset}}_{\mathrm{KSE}}$ is positive:negative=1:2. We observed that with the increasing sampling ratios, the AUPR values decrease, and AUROC values remain almost unchanged. This result suggests that AUPR is an informative metric since it can reveal differences in model performance on balanced or unbalanced datasets. We also observed that KSE performs best on ${\mathrm{Dataset}}_{\mathrm{KSE}}$ (Fig. 5A and B), and KS performs best on ${\mathrm{Dataset}}_{\mathrm{KS}}$ (Fig. 5C and D) under different sampling ratios. Multimodality fusion can significantly improve model performances in the case of sample imbalance. For example, the AUPR of KSE is 8% higher than S (*P *=* *.002). The fusion models KSE, KE, KS, and SE outperform single models K, S, and E when the data volume of negative samples is much larger than that of positive examples, such as 1:5 and 1:10.

### 3.7 Reverse screening and molecular docking

Discovering new DTIs is a crucial step in drug repurposing/discovery. We designed a reverse screening process that employs MDTips to identify candidate targets of the input drug (Fig. 7A). We screened 6766 drugs in ${\mathrm{Dataset}}_{\mathrm{KS}}$ using the KS model, and 857 drugs in ${\mathrm{Dataset}}_{\mathrm{KSE}}$ using the KSE model. We assumed DTIs with a score >0.7 as a credible prediction.

We collected the top 10 credible targets of all the 6766 drugs, involving 2400 targets (Supplementary Table S3). The results show an incredibly high frequency of cytochromes P450 (CYP) family members. The top frequent CYP3A4 appears 2065 times that far more than the runner-up ALB with 1582 counts, meaning that around 30.6% of the drugs are predicted to interact with CYP3A4. CYP family members are heavily involved in drug metabolism (Zanger and Schwab 2013) and should be an essential consideration in drug development. Other high-frequency targets are shown in Supplementary Fig. S2. Several targets play a significant role in anticancer therapy. For example, CDK2 is a druggable target involved in acute myeloid leukemia differentiation and various cancers (e.g. ovarian and breast cancer) (Tadesse *et al.* 2020, Wang *et al.* 2021). CA2 is upregulated in cancers (e.g. hepatocellular carcinoma) (Xing *et al.* 2021) and plays a role in the establishment of tumor endothelium (Annan *et al.* 2019).

**Figure 6.**
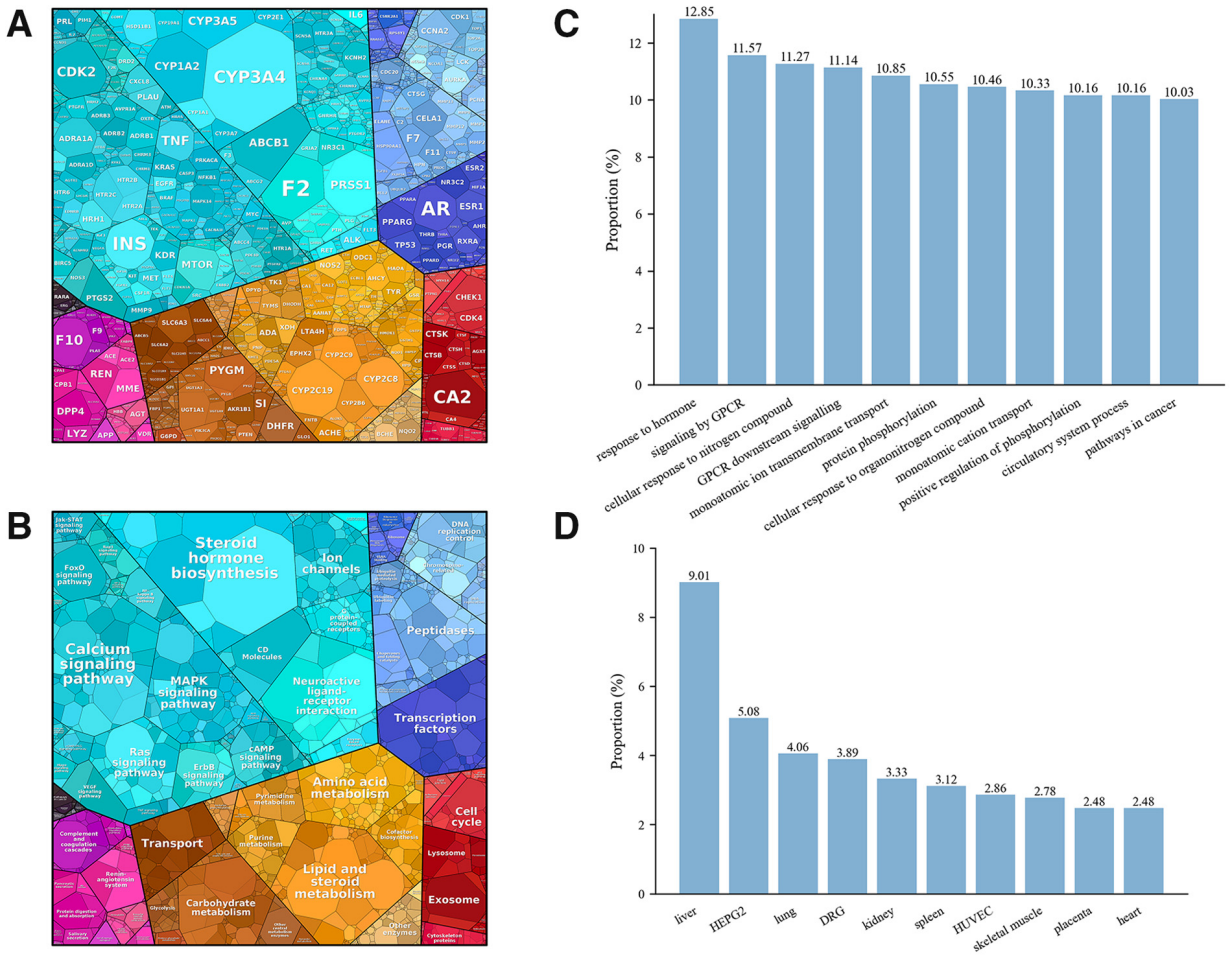
Function analysis of targets. The candidate targets are visualized by Proteomaps (A) Level 5 and (B) Level 3. Top proportion categories in (C) pathway and process, and (D) tissue/cell-specific gene enrichment analysis of targets of Metascape.

All the high-frequency targets mentioned above significantly appeared in the Proteomaps (Liebermeister *et al.* 2014) at the protein level (Fig. 6A). The candidate targets can be further grouped into highly enriched functional categories and pathways, including steroid hormone biosynthesis, amino acid metabolism, and ion channels (Fig. 6B). From the results of Metascape (Zhou *et al.* 2019), targets that response to hormone make up the category of biological functions with the highest proportion (12.85%) of all the targets. Besides, several categories such as GPCR signaling, response to nitrogen compound, and protein phosphorylation are all over 10% proportion of all the targets (Fig. 6C). Tissue/cell-specific results showed exceptionally high proportion (9.01%) of liver expressed targets, much higher than the runner-up tissue lung (4.06%) (Fig. 6D). This result is consistent with the fact that the majority of drugs are closely related to the conversion and metabolism of the liver (Almazroo *et al.* 2017).

### 3.8 Case study

**Figure 7.**
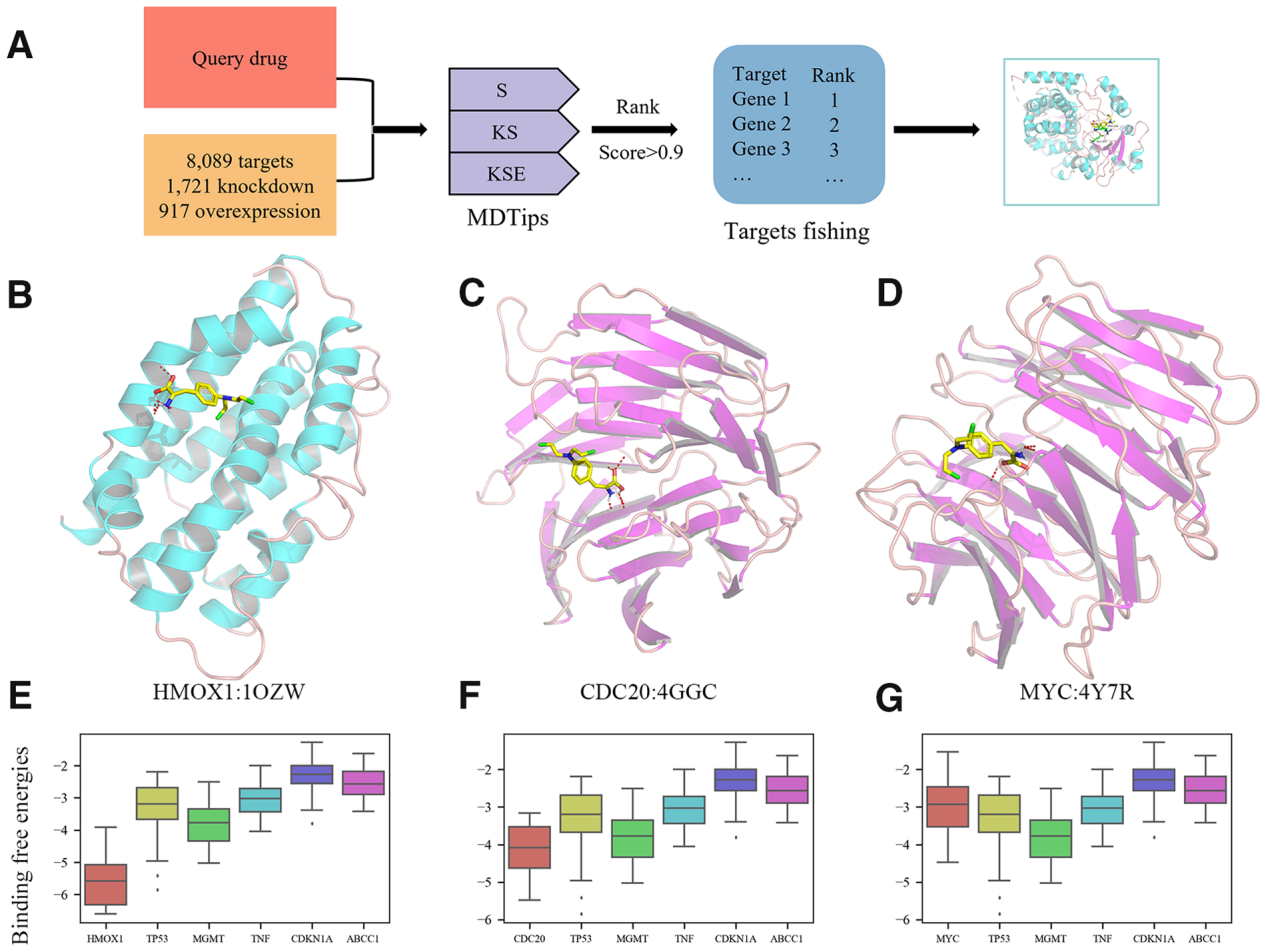
(A) The pipeline of the target prediction of the query drug using the MDTips. (B–D) The bound conformations of melphalan to predicted targets: HMOX1, CDC20, and MYC. (E–G) Compares the binding free energies of melphalan to predicted targets and to the known targets.

Melphalan is a crucial agent in the realm of antineoplastic therapeutics. Its small molecular weight and alkylating properties enable it to interact with multiple targets. Clarification of its putative targets can inform rational structure modifications, augment therapeutic effectiveness, and diminish toxicity. Notably, melphalan possesses three modalities and is the subject of several target investigations. In consideration of this, the KSE and KS models can be employed to prognosticate potential targets (Supplementary Table S4). The results suggest that melphalan may activate HMOX1, inhibit CDC20, and interact with MYC. The molecular docking results show melphalan’s bound conformations (Fig. 7B–D) and binding free energies (Fig. 7E–G) to predicted targets. The lowest binding free energy of melphalan to HMOX1 is −6.61 kcal/mol, significantly lower than that of melphalan to ABCC1 (known target): −3.42 kcal/mol (*P = *7.556×10^−10^).

Moreover, melphalan is predicted to treat cancers, such as breast and ovarian cancer, consistent with previous research that melphalan is a treatment for BRCA-related ovarian carcinoma, breast cancer, and refractory cancer (Bouwman and Jonkers 2012). MDTips also identified the side effects of melphalan, such as nausea, vomiting, and thrombocytopenia (Kashimura *et al.* 1988) (Supplementary Table S5).

## 4 Discussion

Our work still has room for improvement. First, we still need large-scale and highly qualified datasets, which play a critical role in improving the performance of DTI prediction. Although the number of validated DTIs has increased dramatically over the past decades, there still needs to be reliable negative DTI samples. Additionally, many studies focus on hotspot drugs and targets, accumulating extensive interactions of these hotspots. These facts inevitably lead to data bias and poor predictive performance on new targets/drugs that never appeared in the training set. Thus, in this study, we used the known DTIs as much as possible. To our best knowledge, ${\mathrm{Dataset}}_{\mathrm{KS}}$ is the largest DTI dataset, including 88 439 DTIs, 6766 drugs, and 8089 targets. ${\mathrm{Dataset}}_{\mathrm{KSE}}$ is the first large-scale dataset representing drugs and targets with three modalities (i.e. KG, sequence/graph, and expression profiles), including 24 418 DTPs, 857 drugs, and 2108 targets. MDTips covers 8089 targets belonging to one or more of 41 potentially druggable gene categories, according to DGIdb (Freshour *et al.* 2021). The most notably druggable gene categories, such as ion channel and enzyme, are well represented, with 74.1% (352/475) and 70.6% (2194/3106) in MDTips (Supplementary Table S6). However, there still exists inherent biases in the datasets, for hotspot targets such as CYP family members have a relatively high frequency. The fact will inevitably result in the model tending to identify those entities with high frequency.

Second, our current MDTips framework has not yet to consider the 3D structures of drugs and proteins. The result of comparing MDTips to AttentionSiteDTI indicates that DTI models trained on predicted 3D structures do not outperform models trained on AAS. The 3D structure-based models should be trained on highly credible datasets that include measured ligand–receptor affinities and cocrystal structures of ligands and proteins. However, the data volume of these datasets is relatively scarce due to the expensive costs and the need for experimental verification of structures. Recently, AlphaFold2 has been developed to predict structures for most human proteomes based on AAS (Jumper *et al.* 2021) and publicly released high-accuracy protein-structure predictions on the AlphaFold Protein Structure Database (Varadi *et al.* 2022). However, AlphaFold2 has potential limitations in predicting structures of proteins with a small number of experimental structures in the PDB. For example, AlphaFold2 could capture the overall backbone features of the G protein-coupled receptors but fell short in predicting transmembrane domains, the shape of the ligand-binding pockets, and the conformation of the transducer-binding interfaces (He *et al.* 2023), which are critical for interactions with drugs. The application of novel 3D-based methods should rely on credible datasets.

For a fair comparison, all models should be trained on the same dataset. KSE outperformed the other six models trained on the ${\mathrm{Dataset}}_{\mathrm{KSE}}$. Although the LINCS is updating, the data size is relatively small compared with the vast chemical space. The data augmentation method proposed in DeepCE (Pham *et al.* 2021) could solve the problem of limited data.

The common problem of KGE is that the model should be re-trained if new entities or relations are added in, which costs a lot of time and computing resources. This problem still exists in our model. Moreover, it becomes increasingly challenging to re-train KGs with the increasing scale. Transfer learning can solve this problem well (Zhuang *et al.* 2021). For example, entity and relation embeddings of ConvKB are initialized using pretrained parameters produced by TransE (Nguyen *et al.* 2017).

Several potential directions may improve MDTips further: (i) integrating multimodality data with a complex algorithm, such as an attention mechanism, and gaining the interpretability of models will inevitably be the focus of future work. (ii) Learning features from multiple molecular representations by transfer learning is an important research direction. (iii) Incorporating more knowledge into the DTI prediction model with effective KGE methods is also a promising future direction. Therefore, we will integrate more structural representations (sequence strings, molecular graphs, and 3D structures), gene expression profiles, and large-scale KG into the same framework to a further version of MDTips.

## 5 Conclusion

This work has developed a DL-based DTI prediction system: MDTips, which integrates KGs, graphs/sequences, and gene expression signatures to predict the input drug’s potential targets and additional information, such as side effects and indications. We demonstrated that multimodal fusion could significantly improve model performance, and MDTips achieves a high one, indicating KGs and gene expression signatures play a pivotal role in DTI prediction. Additionally, DL-based encoders that Attentive FP and Transformer outperform traditional chemical descriptors/fingerprints and biological features. In addition, MDTips outperforms other state-of-the-art methods, including knowledge-based, sequence-based, graph-based, and 3D structure-based ones. Furthermore, the case study shows that MDTips can predict DTI and additional information such as side effects and indications of the input drug. In summary, MDTips will offer a highly competitive approach for DTI prediction and benefit drug repurposing.

## Supplementary Material

btad411_Supplementary_Data

## Data Availability

The data underlying this article are available in github and zenodo, at https://github.com/XiaoqiongXia/MDTips and https://doi.org/10.5281/zenodo.7560544.
