# Supplementary material for: MDTips: a multimodal-data-based drug–target interaction prediction system fusing knowledge, gene expression profile, and structural data
Source: Bioinformatics. 2023 Jun 28;39(7):btad411. doi: 10.1093/bioinformatics/btad411 (PMC10329491; doi:10.1093/bioinformatics/btad411)
Supplement: btad411_Supplementary_Data [file btad411_supplementary_data.zip › supplementary data.docx]

This supplementary document includes supplementary methods, figures, and tables that support the manuscript “MDTips: A **M**ultimodal-data based **D**rug-**T**arget **i**nteraction **p**rediction **s**ystem fusing knowledge, gene expression profile and structural data”.

# Supplement methods

## Consensus transcriptional profiles

We obtained the consensus transcriptional profiles from https://github.com/dhimmel/lincs. These profiles are based on original transcriptional profiles found in the Expanded CMap LINCS Resource 2020 (Institute, 2020; Subramanian, et al., 2017). Each perturbation in the resource was often assessed across different cell types, dosages, time points, and concentrations, resulting in multiple signatures. To simplify this information, we used consensus transcriptional profiles, which were computed by (Himmelstein, et al., 2017). Consensus profiles meta-analyze multiple signatures and condense them into a single profile for each perturbation.

1. They calculated the weight of each input signature by taking the average Spearman’s correlation with other input signatures and setting a minimum correlation value of 0.05 to ensure all signatures contribute evenly and avoid negative weights. The computation of weights was based only on landmark probes.
2. They employed Stouffer’s method to meta-analyze z-scores and generate a probe-level consensus signature.
3. To obtain a gene-level consensus, they took the average of the probe z-scores for the same Entrez gene and condensed them accordingly.

## GAT

In the messaging phase, GAT is used to aggregate features for $v$ by focusing on its neighbor nodes (i.e., $N(v)$) and local environment (i.e., edge features). Specifically, $C_{v}^{k-1}$ is obtained by the following formulations:

$e_{vu}^{k-1}=leaky\_relu(W[h_{u}^{k-1},h_{v}^{k-1}])$ (1)

$a_{vu}^{k-1}=\mathrm{softmax}\left( e_{vu}^{k-1} \right)=\frac{exp(e_{vu}^{k-1})}{\sum_{u\in N(v)} exp(e_{vu}^{k-1})}$ (2)

$C_{v}^{k-1}=\mathrm{elu}(\sum_{u\in N(v)} a_{vu}^{k-1}Wh_{u}^{k-1})$ (3)

where $[ ]$ is the concatenate operation, $a_{vu}^{k-1}$ is calculated by applying the Softmax function to $e_{vu}^{k-1}$, indicating the importance (weight) of $u$ to $v$.

## Gated Recurrent Unit

Gated recurrent unit (GRU) is used in recursive neural networks. GRU adaptively remembers and forgets information of target atom $v$ from the previous layer with reset gate $r_{v}$ and update gate $z_{v}$, which are formulated as follows:

Reset gate: $r_{v}=\sigma\left( \left[ W_{r}C_{v}^{k-1} \right]+[U_{r}h_{v}^{k-1}] \right)$ (4)

Update gate: $z_{v}=\sigma\left( \left[ W_{z}C_{v}^{k-1} \right]+[U_{z}h_{v}^{k-1}] \right)$ (5)

where $\sigma\left( x \right)=\frac{1}{1+e^{-x}}$ (6)

the output $h_{v}^{k}$ of atom $v$ in the $k-1$ layer is then computed by

$h_{v}^{k}=z_{v}h_{v}^{k-1}+\left( 1-z_{v} \right)\tilde{h_{v}^{k}}$ (7)

where $\tilde{h_{v}^{k}}=\emptyset\left( \left[ WC_{v}^{k-1} \right]+\left[ rh^{k-1} \right] \right)$ (8)

where is element-wise multiplication.

## Multi-head self-attention layer

Firstly, we calculate the attention by

$\mathrm{Attention}(Q,K,V)=\mathrm{softmax}(\frac{QK^{T}}{\sqrt{d}})V$ (9)

where $Q,K,V$ are generated by applying fully connected layers FC to $E$ (e.g., $Q=(EW_{Q}+b_{Q})$). $T$ is a transpose operation. $\frac{1}{\sqrt{d}}$ is a scaled factor. To jointly attend to information from different representation subspaces at different positions, multi-head attention is introduced:

$\mathrm{MultiHead}\left( Q,K,V \right)=\mathrm{Concat}\left( {head}_{1},\ldots,{head}_{h} \right)W^{o}$ (10)

where ${head}_{i}=\mathrm{Attention}(QW_{i}^{Q},KW_{i}^{K},VW_{i}^{V})$ (11)

where $W_{i}^{Q},W_{i}^{K},W_{i}^{V}\epsilon R^{d*d^{'}}$were learnable weight matrices, and $W^{o}\epsilon R^{hd^{'}*d}$. Here, we employ $h=4$ parallel attention heads, $d^{'}=\frac{d}{h}=16$.

## Experimental framework

In order to evaluate the model performance and the minimum impact of data variability, 10-fold cross-validation (10-CV) is used to compare the performance of MDTips and other state-of-the-art methods. The experiment dataset is randomly divided into 10 parts. In each fold, one of the 10 parts is used as the test set, and the remaining 9 parts are randomly split into the training set (8/9) and the validation set (1/9). $\mathrm{KG}_{\sup p\mathrm{ort}}$ and the training set are concatenated to train the KGE model. The validation set is used for early stopping in the training process, and the test set is used for model testing. All models are evaluated on the same training, validation, and test sets.

## Evaluation metrics

In this study, we use the Area under the Receiver Operating Characteristics (AUROC) and the Area Under the Precision-Recall (AUPR) curves to evaluate the performance of each method. The AUPR is more credible when negative samples are much larger than positive ones (Saito and Rehmsmeier, 2015). Thus, we use AUPR as the primary metric and AUROC as the supplementary metric. Furthermore, we use the two-sided Wilcoxon rank sum test with a significance threshold of 0.05 to demonstrate the significant performance difference between MDTips and compared methods. We reported the metrics’ mean and standard deviation by executing 10-CV for each method.

## Early stop strategy

The early stopping strategy is also called “no-improvement-in-*n*”, where *n* is the number of epochs. Early stopping is set during training to avoid overfitting and reduce training time. In the training process, we set a maximum epoch of 100. At the end of each epoch, the AUROC of the current model is calculated using the validation set. The best model is updated if the current AUROC is higher than the highest AUROC. Otherwise, the previous best model is kept. The training process is terminated early if the AUROC has not improved in 5 consecutive epochs on the validation set. When training the final prediction models, we terminate the training process by setting the epoch at *n*, calculated by averaging the training epochs that early stopped with validation datasets in 10-CV.

## Deep learning implementation details

All models are implemented with Pytorch deep learning framework. The training epoch is set to 100 for all datasets and all models. We use Adam optimizer (Kingma and Ba, 2014) with a learning rate of 0.001, BCELoss as a loss function, and the batch size set at 128. The dropout rate is set at 0.1. The validation set is used for early stopping in the training process. GNN models are implemented in DGL-LifeSci. KGE models are implemented in pykeen (Ali, et al., 2021). DeepDTI, GraphDTA, and CPI-GNN models are constructed with DeepPurpose (Huang, et al., 2020). ESPF of targets can be found at <https://github.com/kexinhuang12345/ESPF/tree/master/info>. The consensus transcriptional profiles can be downloaded at https://github.com/dhimmel/lincs. All experiments are conducted on Tesla A100 GPU with 40 GB. For details, please see the website: https://github.com/XiaoqiongXia/MDTips. Data used for training MDTips can be downloaded at <https://doi.org/10.5281/zenodo.7560544>.

## Molecular docking and visualization

AutoDock4 predicts binding free energies and bound conformations of small molecules to macromolecular targets (Morris, et al., 2009). The docking protocol is as follows (Forli, et al., 2016):

1. Download coordinate files for receptors and ligands from PDB (Burley, et al., 2019) and DrugBank, then prepare PDBQT files with AutoDockTools-1.5.7.
2. Pre-calculate atomic affinities using AutoGrid.
3. Docking using AutoDock. The number of requested GA dockings sets to 50.
4. Visualize results using PyMOL.

Proteomaps is used to inspect the categories of targets at different granularity levels (Liebermeister, et al., 2014), and Metascape is used for pathway and process enrichment analysis (Zhou, et al., 2019).

## 6 state-of-the-art models

- **Knowledge-based methods**

1. KGE_NFM (Ye, et al., 2021) first learns knowledge graph embeddings for drugs and targets by DistMult and then integrates structural information: molecular fingerprints and protein descriptors via a neural factorization machine (NFM) for DTI predictions.
2. DRKG (Ioannidis, 2020) is a comprehensive biological knowledge graph. We train a KGE model to obtain the embedding of drugs and targets used for DTI predictions.

- **Sequence-based model**

1. DeepDTA (Öztürk, et al., 2018) models protein amino acid sequences and compound 1D representations with CNNs to predict DTI.

- **Graph-based models**

1. CPI-GNN (Tsubaki, et al., 2019) is an end-to-end DTI model that uses a GNN for compounds and a CNN for proteins.
2. GraphDTA (Nguyen, et al., 2021)represents drugs as graphs and uses graph neural networks (GCN, GIN, and GAT) to predict drug-target interactions.

- **3D structure-based model**

1. AttentionSiteDTI (Yazdani-Jahromi, et al., 2022) is an interpretable graph-based deep learning prediction model which utilizes protein 3D structural information to predict DTI.

# Supplement tables

Table S1. Initial atomic features.

| **Atom feature** | **Size** | **Description** |
| --- | --- | --- |
| atom symbol | 16 | [B, C, N, O, F, Si, P, S, Cl, As, Se, Br, Te, I, At, metal] |
| degree | 6 | number of covalent bonds [0,1,2,3,4,5] |
| formal charge | 1 | electrical charge |
| radical electrons | 1 | number of radical electrons |
| hybridization | 6 | [sp, sp2, sp3, sp3d, sp3d2, other] |
| aromaticity | 1 | whether the atom is part of an aromatic system [0/1] |
| hydrogens | 5 | number of connected hydrogens [0,1,2,3,4] |
| chirality | 1 | whether the atom is chiral center [0/1] |
| chirality type | 2 | [R, S] |

Table S2. Initial bond features.

| **Bond feature** | **Size** | **Description** |
| --- | --- | --- |
| bond type | 4 | [single, double, triple, aromatic] |
| conjugation | 1 | whether the bond is conjugated [0/1] |
| ring | 1 | whether the bond is in ring [0/1] |
| stereo | 4 | [StereoNone, StereoAny, StereoZ, StereoE] |

Table S3. The predicted targets (top 10 & score>0.7) of 6746 drugs.

Table S4. Predicted targets for melphalan with MDTips.

Table S5. Predicted indications, side effects, and drugs for melphalan with MDTips.

| Relation | Predicted result |
| --- | --- |
| drug-disease | breast cancer |
|  | hematologic cancer |
|  | brain cancer |
|  | prostate cancer |
|  | kidney cancer |
|  | peripheral nervous system neoplasm |
|  | ovarian cancer |
|  | multiple sclerosis |
|  | colon cancer |
|  | esophageal cancer |
| drug-side effects | nausea |
|  | vomiting |
|  | myelosuppression |
|  | decreased appetite |
|  | diarrhoea |
|  | body temperature increased |
|  | thrombocytopenia |
|  | dermatitis |
|  | rash |
|  | alopecia |
| drug-drug | fludarabine |
|  | decitabine |
|  | cytarabine |
|  | raltitrexed |
|  | nelarabine |
|  | melphalan |
|  | estramustine |
|  | cladribine |
|  | phenylalanine |
|  | etoposide |

Table S6. The Intersection of target and druggable gene categories in DGIdb

| **Category** | **Gene number** | **Intersection** | **Fraction** |
| --- | --- | --- | --- |
| PHOSPHATIDYLINOSITOL 3 KINASE | 24 | 23 | 0.958 |
| DRUG RESISTANCE | 124 | 116 | 0.935 |
| TYROSINE KINASE | 127 | 117 | 0.921 |
| DNA DIRECTED RNA POLYMERASE | 6 | 5 | 0.833 |
| SERINE THREONINE KINASE | 438 | 358 | 0.817 |
| ABC TRANSPORTER | 46 | 36 | 0.783 |
| SHORT CHAIN DEHYDROGENASE REDUCTASE | 53 | 40 | 0.755 |
| ION CHANNEL | 475 | 352 | 0.741 |
| CYTOCHROME P450 | 57 | 42 | 0.737 |
| HISTONE MODIFICATION | 15 | 11 | 0.733 |
| ENZYME | 3106 | 2194 | 0.706 |
| NUCLEAR HORMONE RECEPTOR | 170 | 120 | 0.706 |
| TRANSCRIPTION FACTOR BINDING | 67 | 47 | 0.701 |
| CLINICALLY ACTIONABLE | 1052 | 732 | 0.696 |
| HORMONE ACTIVITY | 98 | 66 | 0.673 |
| TRANSPORTER | 943 | 632 | 0.670 |
| DNA REPAIR | 232 | 150 | 0.647 |
| TUMOR SUPPRESSOR | 229 | 146 | 0.638 |
| GROWTH FACTOR | 162 | 103 | 0.636 |
| CELL SURFACE | 642 | 405 | 0.631 |
| KINASE | 1938 | 1187 | 0.612 |
| NEUTRAL ZINC METALLOPEPTIDASE | 56 | 34 | 0.607 |
| LIPID KINASE | 5 | 3 | 0.600 |
| DRUGGABLE GENOME | 5776 | 3343 | 0.579 |
| PROTEIN PHOSPHATASE | 109 | 60 | 0.550 |
| TRANSCRIPTION FACTOR COMPLEX | 206 | 108 | 0.524 |
| PROTEASE | 587 | 304 | 0.518 |
| EXTERNAL SIDE OF PLASMA MEMBRANE | 497 | 250 | 0.503 |
| LIPASE | 10 | 5 | 0.500 |
| PROTEASE INHIBITOR | 154 | 76 | 0.493 |
| PHOSPHOLIPASE | 47 | 22 | 0.468 |
| TRANSCRIPTION FACTOR | 1340 | 626 | 0.467 |
| THIOREDOXIN | 30 | 14 | 0.467 |
| METHYL TRANSFERASE | 57 | 26 | 0.456 |
| EXCHANGER | 14 | 6 | 0.429 |
| PTEN FAMILY | 7 | 3 | 0.429 |
| FIBRINOGEN | 32 | 12 | 0.375 |
| MYOTUBULARIN RELATED PROTEIN PHOSPHATASE | 16 | 6 | 0.375 |
| G PROTEIN COUPLED RECEPTOR | 924 | 267 | 0.289 |
| B30_2 SPRY DOMAIN | 89 | 23 | 0.258 |
| RNA DIRECTED DNA POLYMERASE | 10 | 1 | 0.100 |

# Supplement figures


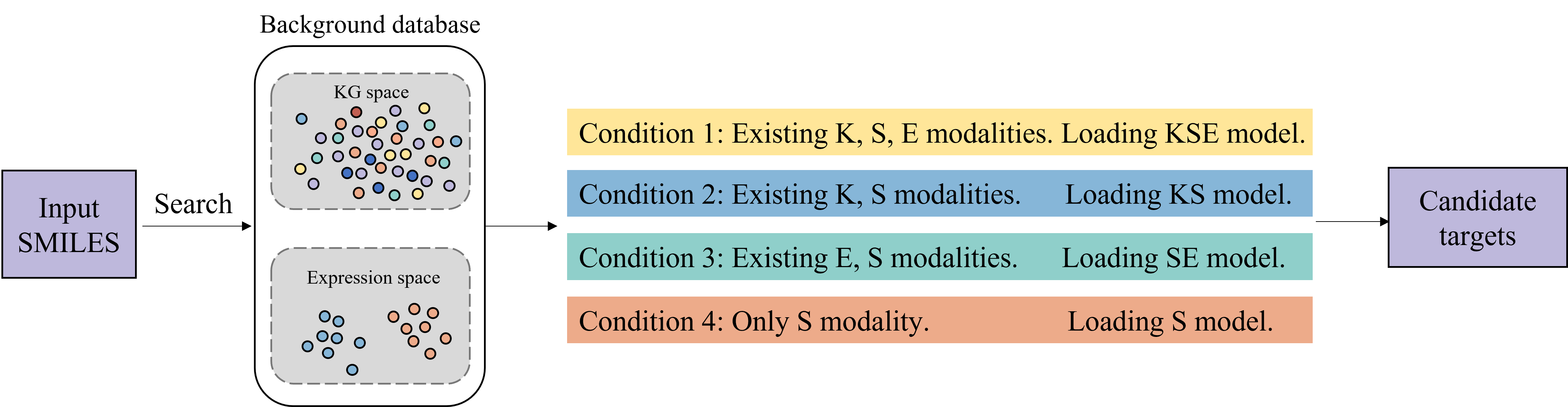


**Fig. S1.** The pipeline of predicting DTI using MDTips.


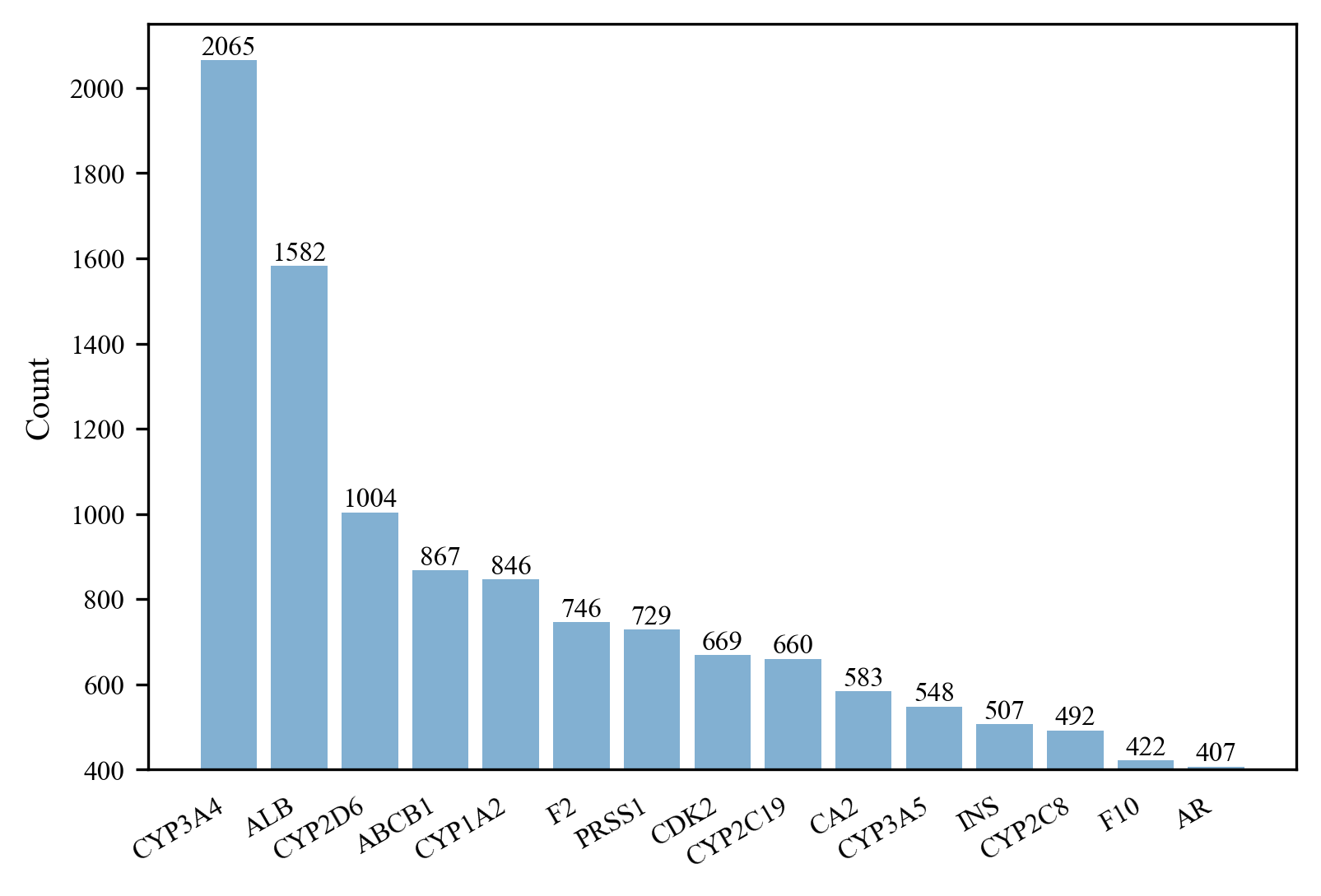


**Fig. S2**. The top 15 targets with high frequency.

# References

Ali, M.*, et al.* Bringing Light Into the Dark: A Large-scale Evaluation of Knowledge Graph Embedding Models under a Unified Framework. *IEEE Transactions on Pattern Analysis and Machine Intelligence* 2021:1-1.

Burley, S.K.*, et al.* RCSB Protein Data Bank: biological macromolecular structures enabling research and education in fundamental biology, biomedicine, biotechnology and energy. *Nucleic acids research* 2019;47(D1):D464-D474.

Forli, S.*, et al.* Computational protein-ligand docking and virtual drug screening with the AutoDock suite. *Nature Protocols* 2016;11(5):905-919.

Himmelstein, D.S.*, et al.* Systematic integration of biomedical knowledge prioritizes drugs for repurposing. *Elife* 2017;6.

Huang, K.*, et al.* DeepPurpose: a deep learning library for drug–target interaction prediction. *Bioinformatics* 2020;36(22-23):5545-5547.

Institute, B. Connectivity Map. 2020. <https://clue.io>. 2020.

Ioannidis, V.N., Song et al. DRKG - Drug Repurposing Knowledge Graph for Covid-19. 2020.

Kingma, D.P. and Ba, J.J.a.e.-p. Adam: A Method for Stochastic Optimization. In.; 2014. p. arXiv:1412.6980.

Liebermeister, W.*, et al.* Visual account of protein investment in cellular functions. *P Natl Acad Sci USA* 2014;111(23):8488-8493.

Morris, G.M.*, et al.* AutoDock4 and AutoDockTools4: Automated Docking with Selective Receptor Flexibility. *Journal of Computational Chemistry* 2009;30(16):2785-2791.

Nguyen, T.*, et al.* GraphDTA: predicting drug-target binding affinity with graph neural networks. *Bioinformatics* 2021;37(8):1140-1147.

Öztürk, H., Özgür, A. and Ozkirimli, E. DeepDTA: deep drug–target binding affinity prediction. *Bioinformatics* 2018;34(17):i821-i829.

Saito, T. and Rehmsmeier, M. The Precision-Recall Plot Is More Informative than the ROC Plot When Evaluating Binary Classifiers on Imbalanced Datasets. *Plos One* 2015;10(3).

Subramanian, A.*, et al.* A Next Generation Connectivity Map: L1000 Platform and the First 1,000,000 Profiles. *Cell* 2017;171(6):1437-1452.e1417.

Tsubaki, M., Tomii, K. and Sese, J. Compound-protein interaction prediction with end-to-end learning of neural networks for graphs and sequences. *Bioinformatics* 2019;35(2):309-318.

Yazdani-Jahromi, M.*, et al.* AttentionSiteDTI: an interpretable graph-based model for drug-target interaction prediction using NLP sentence-level relation classification. *Briefings in Bioinformatics* 2022;23(4).

Ye, Q.*, et al.* A unified drug–target interaction prediction framework based on knowledge graph and recommendation system. *Nature Communications* 2021;12(1):6775.

Zhou, Y.*, et al.* Metascape provides a biologist-oriented resource for the analysis of systems-level datasets. *Nat Commun* 2019;10(1):1523.
